# A new multi-step technique with differential transform method for analytical solution of some nonlinear variable delay differential equations

**DOI:** 10.1186/s40064-016-3386-8

**Published:** 2016-10-06

**Authors:** Brahim Benhammouda, Hector Vazquez-Leal

**Affiliations:** 1 Higher Colleges of Technology, Abu Dhabi Men’s College, P.O. Box 25035, Abu Dhabi, United Arab Emirates; 2 Facultad de Instrumentación, Electrónica, Universidad Veracruzana, Cto. Gonzalo Aguirre Beltrán S/N, 91000 Xalapa, Veracruz Mexico

**Keywords:** Nonlinear variable delay differential equations, Differential transform, Laplace–Padé method, 34-xx, 41A21, 741H10

## Abstract

This work presents an analytical solution of some nonlinear delay differential equations (DDEs) with variable delays. Such DDEs are difficult to treat numerically and cannot be solved by existing general purpose codes. A new method of steps combined with the differential transform method (DTM) is proposed as a powerful tool to solve these DDEs. This method reduces the DDEs to ordinary differential equations that are then solved by the DTM. Furthermore, we show that the solutions can be improved by Laplace–Padé resummation method. Two examples are presented to show the efficiency of the proposed technique. The main advantage of this technique is that it possesses a simple procedure based on a few straight forward steps and can be combined with any analytical method, other than the DTM, like the homotopy perturbation method.

## Background

Differential equations are relevant tools to model a wide variety of physical phenomena across all areas of applied sciences and engineering. Analytical techniques are applied to find the exact solutions of some cases of differential equations; nevertheless, when the differential equations are nonlinear there are no general techniques of solutions. Therefore, numerical methods are important tools to study and understand the quantitative behavior of the nonlinear differential equations with unknown exact solutions. However, such methods can exhibit numerical instabilities, oscillations or false equilibrium states, among others (Gumel 2002, 2003; de Markus and Mickens 1999). This means that the numerical solution may not correspond to the real solution of the original problem. This situation is further aggravated for some types of differential equations like differential-algebraic equations, fractional differential equations and time delay differential equations (DDEs), among others (Ford and Wulf 2000; Engelborghs et al. 2000; Ascher and Petzold 1998; Campbell et al. 2008; Wanner and Hairer 1998).

Time DDEs appear in propagation and transport phenomena, population dynamics, bioscience problems, neural network model, control problems, electrical networks containing lossless transmission lines and economical systems where decisions and effect are separated by some time intervals, among many other applications (Martín and García 2002a, b; Aiello and Freedman 1990; Gourley and Kuang 2004a, b; Kuang 1993; Li et al. 2006; Li and Jiang 2013; Zhang and Zhang 2013). Given the importance of this kind of equations, some numerical methods have been developed to solve them; among them one can mention: variable multi-step methods (Martín and García 2002a, b), Chebyshev polynomials for pantograph differential equation (Sedaghat et al. 2012), a spectral Galerkin method for nonlinear delay convection-diffusion reaction equations (Liu and Zhang 2015), power series method (Benhammouda et al. 2014a) and the differential transform method (DTM) (Karako and Bereketoglu 2009).

In this work, we propose some case studies of nonlinear DDEs with variable time delays. For such case studies, there are no known numerical methods available. Therefore, we propose a multi-step method with a modified version of the DTM (Zhou 1986; Keskin et al. 2007; Benhammouda et al. 2014b; Benhammouda and Vazquez-Leal 2015; Biazar and Eslami 2010; Chen and Liu 1998; Ayaz 2004; Kangalgil and Ayaz 2009; Kanth and Aruna 2009; Arikoglu and Ozkol 2007; Chang and Chang 2008; Kanth and Aruna 2008; Lal and Ahlawat 2015; Odibat et al. 2010; El-Zahar 2013; Gökdoğan et al. 2012) in order to find the solutions of variable time DDEs.

In the literature, there are some works reporting variable time delays. For instance, Taylor series method is applied to solve time-dependent stochastic DDEs (Milošević and Jovanović 2011). In addition, a study of asymptotic neutral type differential equations with variable time delay is given in Skvortsova (2015). Besides, a study of asymptotic behavior of first order differential equations with variable delay is presented in Dix (2005). In Ding et al. (2010), some new conditions for the boundness and stability by means of the contraction mapping principle are given for nonlinear scalar DDEs with variable delays. The issue of uniqueness of variable DDEs is investigated in Winston (1970), Eloe et al. (2005), Liu and Clements (2002) and Luo et al. (2013). Finally, a research for the existence of attractors for differential equations with a variable delay is presented in Graef and Qian (2000) and Caraballo et al. (2001).

Nonetheless, in the present study, we propose different types of variable delays in terms of algebraic expressions of the time. What is more, given that the approximate solutions of the DTM are power series solutions, we propose to extend the domain of convergence by using a combined scheme of a multi-step technique and the Laplace–Padé resummation method (Vazquez-Leal and Guerrero 2014; Filobello-Nino et al. 2013; Jiao et al. 2002; Sweilam and Khader 2009; Momani et al. 2009; Khan and Faraz 2011; Momani and Ertürk 2008; Tsai and Chen 2010; Ebaid 2011).

This paper is organized as follows: in “Differential transform method” section, we introduce the basic concept of the DTM. Then, in “Multi-step technique and DTM for nonlinear variable DDEs” section, the proposed multi-step technique with the use of the DTM to deal with variable time DDEs is presented. “Padé approximant” and “Laplace–Padé resummation method” sections are devoted to present the basic concepts of Padé and Laplace–Padé resummation methods. In “Case studies” section, the type of delay differential equations with variable delays under study is presented and solved using the proposed technique. Finally, discussion and conclusions are given in “Discussion” and “Conclusions” sections, respectively.

## Differential transform method

For convenience of the reader, we will give a review of the differential transform method (DTM) (Zhou 1986; Keskin et al. 2007; Benhammouda et al. 2014b; Benhammouda and Vazquez-Leal 2015; Biazar and Eslami 2010; Chen and Liu 1998; Ayaz 2004; Kangalgil and Ayaz 2009; Kanth and Aruna 2009; Arikoglu and Ozkol 2007; Chang and Chang 2008; Kanth and Aruna 2008; Lal and Ahlawat 2015; Odibat et al. 2010; El-Zahar 2013; Gökdoğan et al. 2012). We will also describe the DTM to solve systems of ordinary differential equations.

### **Definition 1**

(Zhou 1986; Keskin et al. 2007) If a function *u*(*t*) is analytical with respect to *t* in the domain of interest, then

1 $$\begin{aligned} U( k) =\frac{1}{k!}\left[ \frac{d^{k}u(t)}{dt^{k}}\right] _{t=t_{0}}, \end{aligned}$$

is the transformed function of *u*(*t*).

### **Definition 2**

(Zhou 1986; Keskin et al. 2007) The differential inverse transforms of the set $\left\{ U( k) \right\} _{k=0}^{n}$ is defined by

2 $$\begin{aligned} u(t)=\text { }\displaystyle {{\sum _{k=0}^{\infty }}}U( k) \left( t-t_{0}\right) ^{k}. \end{aligned}$$

Substituting (1) into (2), we deduce that

3 $$\begin{aligned} u(t)=\displaystyle {{\sum _{k=0}^{\infty }}}\frac{1}{k!}\left[ \frac{d^{k}u(t) }{dt^{k}}\right] _{t=t_{0}}\left( t-t_{0}\right) ^{k}. \end{aligned}$$

From the above definitions, it is easy to see that the concept of the DTM is obtained from the power series expansion. To illustrate the application of the proposed DTM to solve systems of ordinary differential equations, we consider the nonlinear system

4 $$\begin{aligned} \frac{du(t)}{dt}=f\left( u(t),t\right) ,\quad t\ge t_{0}, \end{aligned}$$

where $f\left( u(t),t\right)$ is a nonlinear smooth function.

System (4) is supplied with some initial conditions

5 $$\begin{aligned} u(t_{0})=u_{0}. \end{aligned}$$

The DTM establishes that the solution of (4) can be written as

6 $$\begin{aligned} u(t)=\displaystyle {{\sum _{k=0}^{\infty }}}U( k) \left( t-t_{0}\right) ^{k}, \end{aligned}$$

where $U(0), U(1),U(2),\ldots$ are unknowns to be determined by the DTM.

Applying the DTM to the initial conditions (5) and system (4) respectively, we obtain the transformed initial conditions

7 $$\begin{aligned} U(0) =u_{0}, \end{aligned}$$

and the recursion system

8 $$\begin{aligned} (1+k)U\left( k+1\right) =F\left( U(0), \ldots , U( k),k\right) ,\quad k=0,1,2, \ldots \end{aligned}$$

where $F\left( U(0), \ldots , U( k),k\right)$ is the differential transforms of $f\left( u(t),t\right)$.

Using (7) and (8), the unknowns $U(k), k=0,1,2, \ldots$ can be determined. Then, the differential inverse transformation of the set of values $\left\{ U(k) \right\} _{k=0}^{m}$ gives the approximate solution

9 $$\begin{aligned} u(t)=\displaystyle {{\sum _{k=0}^{m}}}U(k) (t-t_{0})^{k}, \end{aligned}$$

where *m* is the approximation order of the solution. The exact solution of problem (4)–(5) is then given by (6).

If *U*(*k*) and *V*(*k*) are the differential transforms of *u*(*t*) and *v*(*t*) respectively, then the main operations of the DTM are shown in Table 1.

**Table 1:** Main operations of the DTM

Function	Differential transform
$\alpha u(t)\pm \beta v(t)$	$\alpha U( k) \pm \beta V( k)$
*u*(*t*)*v*(*t*)	${{\sum _{r=0}^{k}}}U\left( r\right) V\left( k-r\right)$
*u*(*t*)*v*(*t*) *w*(*t*)	${{\sum _{r=0}^{k}\sum _{l=0}^{r}}}U\left( l\right) V\left( r-l\right) W\left( k-r\right)$
$\dfrac{d^{n}}{dt^{n}}[u(t) ]$	$\left( k+1\right) \ldots \left( k+n\right) U\left( k+n\right)$
$e^{\lambda t}$	$\dfrac{\lambda ^{k}e^{\lambda t_{0}}}{k!}$
$\sin \left( \omega t\right)$	$\dfrac{\omega ^{k}}{k!}\sin \left( \omega t_{0}+\dfrac{\pi k}{2}\right)$
$\cos \left( \omega t\right)$	$\dfrac{\omega ^{k}}{k!}\cos \left( \omega t_{0}+\dfrac{\pi k}{2}\right)$

The process of the DTM can be described as:

1. Apply the differential transform to the initial conditions (5).
2. Apply the differential transform to the differential system (4) to obtain a recursion system for the unknowns $U(0), U(1),U(2) \ldots$
3. Use the transformed initial conditions (7) and the recursion system (8) to determine the unknowns $U(0),U(1),U(2),\ldots$
4. Use the differential inverse transform formula (9) to obtain an approximate solution for initial-value problem (4)–(5).

The solutions series obtained from the DTM may have limited regions of convergence. Therefore, we propose to apply the Laplace–Padé resummation method (Vazquez-Leal and Guerrero 2014; Filobello-Nino et al. 2013; Jiao et al. 2002; Sweilam and Khader 2009; Momani et al. 2009; Khan and Faraz 2011; Momani and Ertürk 2008; Tsai and Chen 2010; Ebaid 2011) to the DTM truncated series to enlarge the convergence region as depicted in “Padé approximant” and “Laplace–Padé resummation method” sections.

## Multi-step technique and DTM for nonlinear variable DDEs

The type of nonlinear variable delay differential equations (DDEs) which are considered here are given by

10 $$\begin{aligned} u^{\prime }(t)=f\left( u(t),u\left( \lambda t^{n}\right) ,t\right) ,\quad t_{0}\le t\le T, \end{aligned}$$

11 $$\begin{aligned} u(t)=\phi (t),\quad \alpha \le t\le t_{0}, \end{aligned}$$

where the initial condition $\phi (t)$ is given and $\alpha = {\mathop {\mathrm{min}}\nolimits _{t_{0}\le t\le T}}\left( \lambda t^{n}\right) =\lambda t_{0}^{n}, \lambda >0$ and *n* is a positive integer.

For the nonlinear lag function $\theta (t) =\lambda t^{n},$ the delay function is $\tau (t)=t-\lambda t^{n}$ and is such that $0\le \tau (t)\le t$. The solution of (10)–(11) is assumed to exist, unique and analytical.

To solve (10)–(11), we start by finding the first interval of approximation. This is achieved by solving the inequalities $\tau (t) \ge t-t_{0}$ and $t\ge t_{0}$ which lead to the interval $t_{0}\le t\le t_{1}$, where $t_{1}=\left( t_{0}/\lambda \right) ^{1/n}.$ Therefore, the first sub-problem to solve is given by

12 $$\begin{aligned} u^{\prime }(t)=f\left( u(t),u\left( \lambda t^{n}\right) ,t\right) ,\quad t\in I_{0}:=[t_{0},t_{1}], \end{aligned}$$

13 $$\begin{aligned} u(t)=\phi (t),\quad t\in J_{0}:=[\alpha ,t_{0}]. \end{aligned}$$

Since $\lambda t^{n}\in J_{0}$ for $t\in I_{0},$ then substituting (13) into (12) reduces problem (12)–(13) to the following initial-value problem

14 $$\begin{aligned} u_{0}^{\prime }(t)= f\left( u_{0}(t),\phi (\lambda t^{n}),t\right) ,\quad t\in I_{0}, \end{aligned}$$

15 $$\begin{aligned} u_{0}(t_{0})= \phi \left( t_{0}\right) , \end{aligned}$$

where (15) is obtained from (11).

To solve (14)–(15), the differential transform is applied to it to get the recursion

16 $$\begin{aligned} (k+1)U_{0}(k+1)= F\left( U_{0}(0), \ldots , U_{0}(k),k\right) ,\quad k=0,1,2, \, \ldots \end{aligned}$$

17 $$\begin{aligned} U_{0}(0)= \phi \left( t_{0}\right) , \end{aligned}$$

where $F\left( U_{0}(0), \ldots , U_{0}( k),k\right)$ is the differential transform of $f(u_{0}(t),\phi (\lambda t^{n}),t)$.

Using (16)–(17), the unknowns $U_{0}(k)$, for $k=0,1,2, \ldots$ can be determined. Then, the differential inverse transformation of the set of values $\left\{ U_{0}(k) \right\} _{k=0}^{m}$ gives the approximate solution

18 $$\begin{aligned} u_{0}(t)=\displaystyle {{\sum _{k=0}^{m}}}U_{0}( k) \left( t-t_{0}\right) ^{k},\quad t\in I_{0}. \end{aligned}$$

Now if $t_{1}\ge T$, then the above process is terminated and $u_{0}(t)$ is the approximate solution. Otherwise, if $t_{1}<T$, then the inequalities $\tau (t) \ge t-t_{1}$ and $t\ge t_{1}$ are solved to get $t_{2}=\left( t_{1}/\lambda \right) ^{1/n}$. Then, the solution is extended by solving the following sub-problem

19 $$\begin{aligned} u^{\prime }(t)&=f\left( u(t),u\left( \lambda t^{n}\right) ,t\right) ,\quad t\in I_{1}:=[t_{1},t_{2}], \end{aligned}$$

20 $$\begin{aligned} u(t)&=\phi (t),\quad t\in J_{1}:=[t_{0},t_{1}]. \end{aligned}$$

Since $\lambda t^{n}\in J_{1}$ for $t\in I_{1},$ then substituting (20) into (19) reduces problem (19)–(20) to the following initial-value problem

21 $$\begin{aligned} u_{1}^{\prime }(t)=  f\left( u_{1}(t),\phi (\lambda t^{n}),t\right) ,\quad t\in I_{1}, \end{aligned}$$

22 $$\begin{aligned} u_{1}(t_{1})=  \phi \left( t_{1}\right) , \end{aligned}$$

where (22) is obtained from (20).

To solve (21)–(22), the differential transform is applied to it to get the recursion

23 $$\begin{aligned} (k+1)U_{1}(k+1)=  F\left( U_{1}(0), \ldots , U_{1}(k),k\right) ,\quad k=0,1,2, \ldots \end{aligned}$$

24 $$\begin{aligned} U_{1}(0)=  \phi \left( t_{1}\right) , \end{aligned}$$

where $F\left( U_{1}(0), \ldots , U_{1}(k),k\right)$ is the differential transforms of $f\left( u_{1}(t),\phi \left( \lambda t^{n}\right) ,t\right)$.

Using (23)–(24), the unknowns $U_{1}( k), k=0,1,2, \ldots$ can be determined. Then, the differential inverse transformation of the set of values $\left\{ U_{1}( k) \right\} _{k=0}^{m}$ gives the approximate solution

25 $$\begin{aligned} u_{1}(t)=\displaystyle {{\sum _{k=0}^{m}}}U_{1}( k) \left( t-t_{1}\right) ^{k},\quad t\in I_{1}. \end{aligned}$$

Finally, continuing this process, one can extend the domain of the solution to the desired interval.

## Padé approximant

Given an analytical function *u*(*t*) with Maclaurin’s expansion

26 $$\begin{aligned} u(t) =\sum _{n=0}^{\infty }u_{n}t^{n},\quad 0\le t\le T\text {. } \end{aligned}$$

The Padé approximant to *u*(*t*) of order [*L*, *M*] which we denote by $$[L/M]_{u}(t) $$ is defined by (Baker and Graves-Morris 1996)

27 $$\begin{aligned} \left[ L/M\right] _{u}(t) =\frac{p_{0}+p_{1}t+\cdots +p_{L}t^{L} }{1+q_{1}t+\cdots +q_{M}t^{M}}, \end{aligned}$$

where we considered $q_{0}=1$, and the numerator and denominator have no common factors.

The numerator and the denominator in (27) are constructed so that *u*(*t*) and $$[L/M]_{u}(t) $$ and their derivatives agree at $$t=0$$ up to $$L+M$$. That is

28 $$\begin{aligned} u(t)-\left[ L/M\right] _{u}(t) =O\left( t^{L+M+1}\right) . \end{aligned}$$

From (28), we have

29 $$\begin{aligned} u(t) \sum _{n=0}^{M}q_{n}t^{n}-\sum _{n=0}^{L}p_{n}t^{n}=O\left( t^{L+M+1}\right) . \end{aligned}$$

From (29), we get the following algebraic linear systems

30 $$\begin{aligned} \left\{ \begin{array}{l} u_{L}q_{1}+\cdots +u_{L-M+1}q_{M}=-u_{L+1} \\ u_{L+1}q_{1}+\cdots +u_{L-M+2}q_{M}=-u_{L+2} \\ \vdots \\ u_{L+M-1}q_{1}+\cdots +u_{L}q_{M}=-u_{L+M}, \end{array} \right. \end{aligned}$$

and

31 $$\begin{aligned} \left\{ \begin{array}{l} p_{0}=u_{0} \\ p_{1}=u_{1}+u_{0}q_{1} \\ \vdots \\ p_{L}=u_{L}+u_{L-1}q_{1}+\cdots +u_{0}q_{L}. \end{array} \right. \end{aligned}$$

From (30), we calculate first all the coefficients $q_{n}, 1\le n\le M$. Then, the coefficients $p_{n}, 0\le n\le L$ are determined from (31).

Note that for a fixed value of $L+M+1$, the error (28) is smallest when the numerator and denominator of (27) have the same degree or when the numerator has degree one higher than the denominator.

## Laplace–Padé resummation method

Several approximate methods provide power series solutions (polynomial). Nevertheless, sometimes, this type of solutions lack large domains of convergence. Therefore, Laplace–Padé resummation method (Vazquez-Leal and Guerrero 2014; Filobello-Nino et al. 2013; Jiao et al. 2002; Sweilam and Khader 2009; Momani et al. 2009; Khan and Faraz 2011; Momani and Ertürk 2008; Tsai and Chen 2010; Ebaid 2011) is used in literature to enlarge the domain of convergence of solutions or inclusive to find the exact solutions.

The Laplace–Padé method can be summarized as follows:

1. First, Laplace transformation is applied to power series (9).
2. Next, *s* is substituted by 1/*t* in the resulting equation.
3. After that, the transformed series is convert into a meromorphic function by forming its Padé approximant of order [*N*/*M*]. *N* and *M* are arbitrarily chosen, but they should be smaller than the order of the power series. In this step, the Padé approximant extends the domain of the truncated series solution to obtain better accuracy and convergence.
4. Then, *t* is substituted by 1/*s*.
5. Finally, by using the inverse Laplace *s* transformation, the exact or an approximate solution is obtained.

## Case studies

In this section, we will demonstrate the effectiveness and accuracy of the multi-step technique proposed in “Multi-step technique and DTM for nonlinear variable DDEs” section with the differential transform method (DTM) (Zhou 1986; Keskin et al. 2007; Benhammouda et al. 2014b; Benhammouda and Vazquez-Leal 2015; Biazar and Eslami 2010; Chen and Liu 1998; Ayaz 2004; Kangalgil and Ayaz 2009; Kanth and Aruna 2009; Arikoglu and Ozkol 2007; Chang and Chang 2008; Kanth and Aruna 2008; Lal and Ahlawat 2015; Odibat et al. 2010; El-Zahar 2013; Gökdoğan et al. 2012) through two nonlinear variable delay differential equations (DDEs).

### Example 1

Consider the following nonlinear variable delay differential equation

32 $$\begin{aligned} u^{\prime }(t) +u(t) -u^{2}(t) +u\left( \dfrac{t^{2}}{4}\right)=  f(t),\quad t_{0}\le t\le 2 \sqrt{2}, \end{aligned}$$

33 $$\begin{aligned} u(t)=  e^{-t}, \quad 1/4\le t\le t_{0}, \end{aligned}$$

where $$f(t) =e^{-\frac{t^{2}}{4}}-e^{-2t}$$ and $$t_{0}=1.$$

For this problem the lag function is $$\theta (t) =t^{2}/4$$ and the variable delay is $$\tau (t) =t-t^{2}/4$$. Following the procedure described in “Multi-step technique and DTM for nonlinear variable DDEs” section, problem (32)–(33) is solved step by step. The first interval of approximation is determined by solving the inequalities $$\tau (t) \ge t-t_{0}$$ and $$t\ge t_{0}$$ which lead to the interval $$t_{0}\le t\le t_{1},$$ where $$ t_{1}=2.$$ Therefore, the first sub-problem to solve is given by

34 $$\begin{aligned} u_{0}^{\prime }(t) +u_{0}(t) -u_{0}^{2}(t) +u_{0}\left( \dfrac{t^{2}}{4}\right)=  f(t), \quad t\in I_{0}:=\left[ t_{0},t_{1}\right] , \end{aligned}$$

35 $$\begin{aligned} u_{0}(t)=  e^{-t}, \quad t\in J_{0}:=\left[ 1/4,t_{0} \right] . \end{aligned}$$

Since $t^{2}/4\in J_{0}$ for $t\in I_{0},$ then substituting (35) into (34) reduces problem (34)–(35) into the following initial-value problem

36 $$\begin{aligned} u_{0}^{\prime }(t) +u_{0}(t) -u_{0}^{2}(t)=  -e^{-2t}, \quad t\in I_{0}, \end{aligned}$$

37 $$\begin{aligned} u_{0}\left( t_{0}\right)=  e^{-t_{0}}, \end{aligned}$$

where condition (37) is obtained from (33).

To solve (36)–(37), the differential transform is applied to it to obtain the following recursion

38 $$\begin{aligned} \left( k+1\right) U_{0}\left( k+1\right) +U_{0}( k) -{{ \sum _{l=0}^{k}}}U_{0}\left( l\right) U_{0}\left( k-l\right)=  -\frac{\left( -2\right) ^{k}}{k!}e^{-2}, \end{aligned}$$

39 $$\begin{aligned} U_{0}(0)=  e^{-t_{0}},\quad k=0,1,2,\ldots \end{aligned}$$

From recursion (38)–(39), the following $U_{0}( k)$ values are obtained

40 $$\begin{aligned} U_{0}(1)=  -e^{-t_{0}},\quad U_{0}(2) =\frac{1 }{2}e^{-t_{0}},\quad U_{0}(3) =-\frac{1}{3!}e^{-t_{0}},\quad U_{0}(4) =\frac{1}{4!}e^{-t_{0}}, \end{aligned}$$

41 $$\begin{aligned} U_{0}(5)=  -\frac{1}{5!}e^{-t_{0}},\quad U_{0}(6) =\frac{1}{6!}e^{-t_{0}},\quad U_{0} (7) =-\frac{1}{7!}e^{-t_{0}},\quad U_{0}( 8) =\frac{1}{ 8!}e^{-t_{0}}. \end{aligned}$$

From these values, an eighth-order approximate solution is constructed

42 $$\begin{aligned} u_{0}(t) ={{\sum _{k=0}^{8}}}U_{0}( k) \left( t-t_{0}\right) ^{k}=e^{-t_{0}}{{\sum _{k=0}^{8}}}\frac{\left( -1\right) ^{k}}{ k!}\eta _{0}^{k}, \end{aligned}$$

where $$\eta _{0}=t-t_{0}.$$

Note that one can use more terms in the above series solutions. Nevertheless, sometimes, this may not increase the accuracy of the solution for large intervals. Therefore, we use the Laplace–Padé resummation method presented in “Laplace–Padé resummation method” section to enlarge the domain of convergence of solutions or to find the exact solutions as follows.

Applying Laplace transform to $$u_{0}(t) $$ yields

43 $$\begin{aligned} {\mathcal {L}}\left[ u_{0}(t) \right] =e^{-t_{0}}\sum _{k=0}^{8} \frac{(-1) ^{k}}{k!s^{k+1}}. \end{aligned}$$

For simplicity let $s=1/\eta _{0}$, then

44 $$\begin{aligned} {\mathcal {L}}\left[ u_{0}(t) \right] =e^{-t_{0}}\sum _{k=0}^{8} \frac{\left( -1\right) ^{k}}{k!}\eta _{0}^{k+1}. \end{aligned}$$

All of the $[L/M] \eta _{0}$-Padé approximants of (44) with $L \ge 1$ and $M \ge 1$ and $L+M\le 9$ yield

45 $$\begin{aligned} \left[ \dfrac{L}{M}\right] (t) =\frac{e^{-t_{0}}\eta _{0}}{ 1+\eta _{0}}. \end{aligned}$$

Now since $\eta _{0}=1/s$, we obtain $\left[ L/M\right] (t)$ in terms of *s* as follows

46 $$\begin{aligned} \left[ \dfrac{L}{M}\right] (t) =\frac{e^{-t_{0}}}{s+1}. \end{aligned}$$

Finally, applying the inverse Laplace transform to (46) gives the following approximate solution

47 $$\begin{aligned} u_{0}(t) =e^{-t_{0}}e^{-\eta _{0}}=e^{-t},\quad t\in I_{0}, \end{aligned}$$

which is the exact solution of delay equation (34)–(35).

In a similar manner, the second interval of approximation is determined. The conditions $\tau (t) \ge t-t_{1}$ and $t\ge t_{1}$ yield the interval $t_{1}\le t\le t_{2}$ where $t_{2}=2\sqrt{2}.$ Therefore, we consider the sub-problem:

48 $$\begin{aligned} u_{1}^{\prime }(t) +u_{1}(t) -u_{1}^{2}(t) +u_{1}\left( \dfrac{t^{2}}{4}\right)=  f(t),\quad t\in I_{1}:=\left[ t_{1},t_{2}\right] , \end{aligned}$$

49 $$\begin{aligned} u_{1}(t)=  u_{0}(t),\quad t\in J_{1}:= \left[ t_{0},t_{1}\right] . \end{aligned}$$

Since $t^{2}/4\in J_{1}$ for $t\in I_{1},$ then substituting (49) into (48) reduces problem (48)–(49) to the following initial-value problem

50 $$\begin{aligned} u_{1}^{\prime }(t) +u_{1}(t) -u_{1}^{2}(t)=  -e^{-2t},\quad t\in I_{1}, \end{aligned}$$

51 $$\begin{aligned} u_{1}\left( t_{1}\right)=  e^{-t_{1}}, \end{aligned}$$

where condition (51) is obtained from (49).

To solve (50)–(51), the differential transform is applied to it to obtain the following recursion

52 $$\begin{aligned} \left( k+1\right) U_{1}\left( k+1\right) +U_{1}( k) -{{ \sum _{l=0}^{k}}}U_{1}(l) U_{1}\left( k-l\right)=  -\frac{\left( -2\right) ^{k}}{k!}e^{-4}, \end{aligned}$$

53 $$\begin{aligned} U_{1}(0)=  e^{-t_{1}}, \quad k=0,1,2,\ldots \end{aligned}$$

From recursion (52)–(53), the following $U_{1}( k)$ values are obtained

54 $$\begin{aligned} U_{1}(1)=  -e^{-t_{1}},\quad U_{1}(2) =\frac{1 }{2}e^{-t_{1}},\quad U_{1}(3) =-\frac{1}{3!}e^{-t_{1}},\quad U_{1}(4) =\frac{1}{4!}e^{-t_{1}}, \end{aligned}$$

55 $$\begin{aligned} U_{1}(5)=  -\frac{1}{5!}e^{-t_{1}},\quad U_{1}(6) =\frac{1}{6!}e^{-t_{1}},\quad U_{1}(7) =-\frac{1}{7!}e^{-t_{1}} , \quad U_{1}(8) =\frac{1}{ 8!}e^{-t_{1}}. \end{aligned}$$

From these values, an eighth-order approximate solution is constructed

56 $$\begin{aligned} u_{1}(t) ={{\sum _{k=0}^{8}}}U_{1}( k) \left( t-t_{1}\right) ^{k}=e^{-t_{1}}{{\sum _{k=0}^{8}}}\frac{\left( -1\right) ^{k}}{ k!}\eta _{1}^{k}, \end{aligned}$$

where $$\eta _{1}=t-t_{1}.$$

Applying Laplace transform to $$u_{1}(t) $$ yields

57 $$\begin{aligned} {\mathcal {L}}\left[ u_{1}(t) \right] =e^{-t_{1}}\sum _{k=0}^{8} \frac{\left( -1\right) ^{k}}{k!s^{k+1}}. \end{aligned}$$

For simplicity let $s=1/\eta _{1}$, then

58 $$\begin{aligned} {\mathcal {L}}\left[ u_{1}(t) \right] =e^{-t_{1}}\sum _{k=0}^{8} \frac{\left( -1\right) ^{k}}{k!}\eta _{1}^{k+1}. \end{aligned}$$

All of the [*L*/*M*] $\eta _{1}$-Padé approximants of (58) with *L* $\ge 1$ and *M* $\ge 1$ and $L+M\le 9$ yield

59 $$\begin{aligned} \left[ \dfrac{L}{M}\right] (t) =\frac{e^{-t_{1}}\eta _{1}}{ 1+\eta _{1}}. \end{aligned}$$

Now since $\eta _{1}=1/s$, we obtain $\left[ L/M\right] (t)$ in terms of *s* as follows

60 $$\begin{aligned} \left[ \dfrac{L}{M}\right] (t) =\frac{e^{-t_{1}}}{s+1}. \end{aligned}$$

Finally, applying the inverse Laplace transform to (60) gives the following approximate solution

61 $$\begin{aligned} u_{1}(t) =e^{-t_{1}}e^{-\eta _{1}}=e^{-t}, \quad t\in I_{1}, \end{aligned}$$

which is the exact solution of delay equation (48)–(49).

Finally, combining (47) and (61), the exact solution $u(t) =e^{-t}, 1/4\le t\le 2\sqrt{2}$ for the nonlinear variable delay problem (32)–(33) is obtained.

### Example 2

Consider the following nonlinear variable delay differential equation

62 $$\begin{aligned} u^{\prime \prime }(t) +2u(t) -u^{2}(t) +u\left( \dfrac{t^{3}}{8}\right)=  g(t),\quad t_{0}\le t\le 2, \end{aligned}$$

63 $$\begin{aligned} u(t)=  \sin t,\quad 8^{-4}\le t\le t_{0}, \end{aligned}$$

where $$g(t) =\sin t-\sin ^{2}t+\sin \left( t^{3}/8\right) $$ and $$ t_{0}=1/8.$$

For this problem the lag function is $\theta (t) =t^{3}/8$ and the delay is $\tau (t) =t-t^{3}/8.$ As for example 1, problem (62)–(63) is solved step by step. The first interval of approximation is determined by solving the inequalities $\tau (t) \ge t-t_{0}$ and $t\ge t_{0}$ which lead to the interval $t_{0}\le t\le t_{1},$ where $t_{1}=1.$ Thus, the first sub-problem to solve is given by:

64 $$\begin{aligned} u_{0}^{\prime \prime }(t) +2u_{0}(t) -u_{0}^{2}(t) +u_{0}\left( \dfrac{t^{3}}{8}\right)=  g(t),\quad t\in I_{0}:=\left[ t_{0},t_{1}\right] , \end{aligned}$$

65 $$\begin{aligned} u_{0}(t)=  \sin t,\quad t\in J_{0}:=\left[ 8^{-4},t_{0} \right] . \end{aligned}$$

Since $t^{3}/8\in J_{0}$ for $t\in I_{0},$ then substituting (65) into (64) reduces problem (64)–(65) to the following initial-value problem

66 $$\begin{aligned} u_{0}^{\prime \prime }(t) +2u_{0}(t) -u_{0}^{2}(t)=  \sin t-\sin ^{2}t,\quad t\in I_{0}, \end{aligned}$$

67 $$\begin{aligned} u_{0}\left( t_{0}\right)=  \sin t_{0},\quad u_{0}^{\prime }\left( t_{0}\right) =\cos t_{0}, \end{aligned}$$

where conditions (67) are obtained from (63).

To solve (66)–(67), the differential transform is applied to it to obtain the following recursion

68 $$\begin{aligned} \left( k+1\right) \left( k+2\right) U_{0}\left( k+2\right) +2U_{0}( k) -{{\sum _{l=0}^{k}}}U_{0}\left( l\right) U_{0}\left( k-l\right) =S(k)-{{\sum _{l=0}^{k}}}S\left( l\right) S\left( k-l\right) , \end{aligned}$$

where $k=0,1,2,\ldots$ and $U_{0}(0) =\sin t_{0}, \quad U_{0}(1) =\cos t_{0}$ and where $S( k) =\left( 1/k!\right) \sin (t_{0}+k\pi /2)$ represents the differential transform of $\sin t$ at $t_{0}.$

From recursion (68), the following $$U_{0}( k) $$ values are obtained

69 $$ U_{0}(2) =  -\frac{1}{2}\sin t_{0}, \quad U_{0}(3) =-\frac{1}{3!}\cos t_{0}, \quad U_{0}(4) =  \frac{1}{ 4!}\sin t_{0}, \quad U_{0}(5) =  \frac{1}{5!}\cos t_{0}, $$

70 $$U_{0}(6) =  -\frac{1}{6!}\sin t_{0}, U_{0}(7) =-\frac{1}{7!}\cos t_{0}, U_{0}(8) = \frac{1}{8!}\sin t_{0}.$$

From these values, an eighth-order approximate solution is constructed

71 $$\begin{aligned} u_{0}(t) & {}=  {{\sum _{k=0}^{8}}}U_{0}( k) \left( t-t_{0}\right) ^{k}=\left( \sin t_{0}\right) {{\sum _{k=0}^{4}}}\frac{(-1)^{k} }{(2k)!}\eta _{0}^{2k}\nonumber \\&\quad+\,\left( \cos t_{0}\right) {{\sum _{k=0}^{3}}}\frac{ (-1)^{k}}{(2k+1)!}\eta _{0}^{2k+1}, \end{aligned}$$

where $$\eta _{0}=t-t_{0}.$$

To enlarge the domain of convergence of the approximate solution or to find the exact solution, the Laplace–Padé resummation method is used as in the previous example.

Applying Laplace transform to $$u_{0}(t) $$ yields

72 $$\begin{aligned} {\mathcal {L}}\left[ u_{0}(t) \right] =\left( \sin t_{0}\right) \sum _{k=0}^{4}\frac{(-1)^{k}}{(2k)!s^{2k+1}}+\left( \cos t_{0}\right) \sum _{k=0}^{3}\frac{(-1)^{k}}{(2k+1)!s^{2k+2}}. \end{aligned}$$

For simplicity let $s=1/\eta _{0}$, then

73 $$\begin{aligned} {\mathcal {L}}\left[ u_{0}(t) \right] =\left( \sin t_{0}\right) \sum _{k=0}^{4}\frac{(-1)^{k}}{(2k)!}\eta _{0}^{2k+1}+\left( \cos t_{0}\right) \sum _{k=0}^{3}\frac{(-1)^{k}}{(2k+1)!}\eta _{0}^{2k+2}. \end{aligned}$$

All of the $[L/M] \eta _{0}$-Padé approximants of (73) with $L \ge 1$ and $M \ge 1$ and $L+M\le 9$ yield

74 $$\begin{aligned} \left[ \dfrac{L}{M}\right] (t) =\frac{\eta _{0}^{2}\cos t_{0}+\eta _{0}\sin t_{0}}{1+\eta _{0}^{2}}. \end{aligned}$$

Now since $\eta _{0}=1/s$, we obtain $\left[ L/M\right] (t)$ in terms of *s* as follows

75 $$\begin{aligned} \left[ \dfrac{L}{M}\right] (t) =\frac{s\sin t_{0}+\cos t_{0}}{ s^{2}+1}. \end{aligned}$$

Finally, applying the inverse Laplace transform to (75) gives the following approximate solution

76 $$\begin{aligned} u_{0}(t) =\sin t_{0}\cos \eta _{0}+\cos t_{0}\sin \eta _{0}=\sin (\eta _{0}+t_{0})=\sin t,\quad t\in I_{0}. \end{aligned}$$

Thus, the exact solution of the nonlinear variable delay equation (64)–(65) is $u_{0}(t) =\sin t, t_{0}\le t\le 1$.

In a similar manner, the second interval of approximation is determined. This interval is obtained by solving the inequalities $\tau (t) \ge t-t_{1}$ and $t\ge t_{1}$ which lead to the interval $t_{1}\le t\le t_{2}$, where $t_{2}=2.$ Thus, the second sub-problem to solve is given by:

77 $$\begin{aligned} u_{1}^{\prime \prime }(t) +2u_{1}(t) -u_{1}^{2}(t) +u_{1}\left( \dfrac{t^{3}}{8}\right)=  g(t),\quad t\in I_{1}:=\left[ t_{1},t_{2}\right] , \end{aligned}$$

78 $$\begin{aligned} u_{1}(t)=  u_{0}(t),\quad t\in J_{1}:= \left[ 1/8,t_{1}\right] . \end{aligned}$$

Since $t^{3}/8\in J_{1}$ for $t\in I_{1},$ then substituting (78) into (77) reduces problem (77)–(78) to the following initial-value problem

79 $$\begin{aligned} u_{1}^{\prime \prime }(t) +2u_{1}(t) -u_{1}^{2}(t)=  \sin t-\sin ^{2}t,\quad t\in I_{1}, \end{aligned}$$

80 $$\begin{aligned} u_{1}\left( t_{1}\right)=  \sin t_{1},\quad u_{1}^{\prime }\left( t_{1}\right) =\cos t_{1}, \end{aligned}$$

where conditions (80) are obtained from (78).

To solve (79)–(80), the differential transform is applied to it to obtain the following recursion

81 $$\begin{aligned}&\left( k+1\right) \left( k+2\right) U_{1}\left( k+2\right) +2U_{1}( k) -{{\sum _{l=0}^{k}}}U_{1}\left( l\right) U_{1}\left( k-l\right) \nonumber \\&\quad =S(k)-{{\sum _{l=0}^{k}}}S\left( l\right) S\left( k-l\right) , \end{aligned}$$

where $k=0,1,2,\ldots$ and $U_{1}(0) =\sin t_{1}, U_{1}(1) =\cos t_{1}$ and where $S( k) =\left( 1/k!\right) \sin (t_{1}+k\pi /2)$ represents the differential transform of $\sin t$ at $t_{1}.$

From recursion (81), the following $$U_{1}( k) $$ values are obtained

82 $$\begin{aligned} U_{1}(2) & =  -\frac{1}{2}\sin t_{1}, \quad U_{1}(3) =-\frac{1}{3!}\cos t_{1}, \nonumber \\ U_{1}(4) &=  \frac{1}{ 4!}\sin t_{1}, \quad U_{1}\left( 5\right) =\frac{1}{5!}\cos t_{1} , \end{aligned}$$

83 $$\begin{aligned} \quad U_{1}(6) &=  -\frac{1}{6!}\sin t_{1}, \quad U_{1}(7) =-\frac{1}{7!}\cos t_{1}, \nonumber \\ U_{1}(8) &=  \frac{1}{8!}\sin t_{1}. \end{aligned}$$

From these values, an eighth-order approximate solution is constructed

84 $$\begin{aligned} u_{1}(t) & =  {{\sum _{k=0}^{8}}}U_{1}(k) \left( t-t_{1}\right) ^{k}=\left( \sin t_{1}\right) {{\sum _{k=0}^{4}}}\frac{(-1)^{k} }{(2k)!}\eta _{1}^{2k}\nonumber \\&\quad+\,\left( \cos t_{1}\right) {{\sum _{k=0}^{3}}}\frac{ (-1)^{k}}{(2k+1)!}\eta _{1}^{2k+1}, \end{aligned}$$

where $$\eta _{1}=t-t_{1}.$$

Applying Laplace transform to $$u_{1}(t) $$ yields

85 $$\begin{aligned} {\mathcal {L}}\left[ u_{1}(t) \right] & =  \left( \sin t_{1}\right) \sum _{k=0}^{4}\frac{\left( -1\right) ^{k}}{(2k)!s^{2k+1}}\nonumber \\&\quad+\,\left( \cos t_{1}\right) \sum _{k=0}^{3}\frac{\left( -1\right) ^{k}}{(2k+1)!s^{2k+2}}. \end{aligned}$$

For simplicity let $s=1/\eta _{1}$, then

86 $$\begin{aligned} {\mathcal {L}}\left[ u_{1}(t) \right] & =  \left( \sin t_{1}\right) \sum _{k=0}^{4}\frac{\left( -1\right) ^{k}}{(2k)!}\eta _{1}^{2k+1}\nonumber \\&\quad+\,\left( \cos t_{1}\right) \sum _{k=0}^{3}\frac{\left( -1\right) ^{k}}{(2k+1)!}\eta _{1}^{2k+2}. \end{aligned}$$

All of the $[L/M] \eta _{1}$-Padé approximants of (86) with $L \ge 1$ and $M \ge 1$ and $L+M\le 9$ yield

87 $$\begin{aligned} \left[ \dfrac{L}{M}\right] (t) =\frac{\eta _{1}^{2}\cos t_{1}+\eta _{1}\sin t_{1}}{1+\eta _{1}^{2}}. \end{aligned}$$

Now since $\eta _{1}=1/s$, we obtain $\left[ L/M\right] (t)$ in terms of *s* as follows

88 $$\begin{aligned} \left[ \dfrac{L}{M}\right] (t) =\frac{s\sin t_{1}+\cos t_{1}}{ s^{2}+1}. \end{aligned}$$

Finally, applying the inverse Laplace transform to (88) gives the following approximate solution

89 $$\begin{aligned} u_{1}(t) =\sin t_{1}\cos \eta _{1}+\cos t_{1}\sin \eta _{1}=\sin (\eta _{1}+t_{1})=\sin t, \quad t\in I_{1}. \end{aligned}$$

Thus, the exact solution of the nonlinear variable delay equation (77)–(78) is $u_{1}(t) =\sin t, t_{0}\le t\le 2.$

Finally, combining (76) and (89), the exact solution $u(t) =\sin t,\quad 8^{-4}\le t\le 2$ of the nonlinear variable delay problem (62)–(63) is obtained.

## Discussion

Variable delays differential equations (DDEs) with arbitrary types of nonlinear functions for the time delay is an open area of research that require new numerical and analytical methods in order to deal with their solution. Therefore, from the examples above, it is worthwhile to remark that the multi-step technique proposed in this work combined with modified differential transform method (DTM) (Zhou 1986; Keskin et al. 2007; Benhammouda et al. 2014b; Benhammouda and Vazquez-Leal 2015; Biazar and Eslami 2010; Chen and Liu 1998; Ayaz 2004; Kangalgil and Ayaz 2009; Kanth and Aruna 2009; Arikoglu and Ozkol 2007; Chang and Chang 2008; Kanth and Aruna 2008; Lal and Ahlawat 2015; Odibat et al. 2010; El-Zahar 2013; Gökdoğan et al. 2012) based on Laplace–Padé resummation method (Vazquez-Leal and Guerrero 2014; Filobello-Nino et al. 2013; Jiao et al. 2002; Sweilam and Khader 2009; Momani et al. 2009; Khan and Faraz 2011; Momani and Ertürk 2008; Tsai and Chen 2010; Ebaid 2011) was able to obtain the exact solutions of nonlinear DDEs with variable delays. It is important to highlight that this multi-step technique was able to obtain the exact solutions for both case studies within the given intervals. The straight forward procedure was able to deal with different algebraic time delays as quadratic and cubic term highlighting the malleability of the technique presented to solve nonlinear DDEs with variable delays.

As far as the knowledge of authors goes, no numerical or analytical approximation methods to solve the type of case studies of this work have been reported in the literature. Hence, we propose as a proof of concept, to solve problems with known exact solutions. Therefore, this multi-step technique in combination with the DTM and Laplace–Padé resummation was able to obtain the exact solutions within the given intervals. However, it is still pending for future work in order to treat DDEs with unknown exact solutions. For such hard to solve problems, we will measure the error of approximation using the mean square residual (MSR) error as in Benhammouda and Vazquez-Leal (2015). Finally, further research is required to extend this proposed methodology to other delay functions and systems of DDEs, delay differential-algebraic equations and delay partial differential equations with variable time delays.

## Conclusions

This article deals with the solution of nonlinear DDEs with variable delays using a new multi-step method and the DTM (Zhou 1986; Keskin et al. 2007; Benhammouda et al. 2014b; Benhammouda and Vazquez-Leal 2015; Biazar and Eslami 2010; Chen and Liu 1998; Ayaz 2004; Kangalgil and Ayaz 2009; Kanth and Aruna 2009; Arikoglu and Ozkol 2007; Chang and Chang 2008; Kanth and Aruna 2008; Lal and Ahlawat 2015; Odibat et al. 2010; El-Zahar 2013; Gökdoğan et al. 2012). This technique was tested on two nonlinear problems. The results obtained show that the technique can be applied to solve these types of equations efficiently obtaining the exact solution. On the one hand, it is important to highlight that this kind of variable delay present series issues for traditional numerical and analytical methods, and on the other, the DTM in combination with Laplace–Padé resummation method (Vazquez-Leal and Guerrero 2014; Filobello-Nino et al. 2013; Jiao et al. 2002; Sweilam and Khader 2009; Momani et al. 2009; Khan and Faraz 2011; Momani and Ertürk 2008; Tsai and Chen 2010; Ebaid 2011) was able to obtain the exact solutions of nonlinear DDEs with variable delays. Future work is necessary to involve into the application of the proposed methodology for the solution of nonlinear DDEs with variable delays and unknown exact solutions. Other types of problems, like delay differential-algebraic equations and delay partial differential equations with variable time delays, will also be considered.
